# Factors that influence the androgen receptor cistrome in benign and malignant prostate cells

**DOI:** 10.1002/1878-0261.12572

**Published:** 2019-10-13

**Authors:** Ben T. Copeland, Juan Du, Sumanta K. Pal, Jeremy O. Jones

**Affiliations:** ^1^ Deparment of Medical Oncology City of Hope Duarte CA USA; ^2^ Integrative Genomics Core City of Hope Duarte CA USA

**Keywords:** androgen, cancer, cistrome, malignancy, prostate, receptor

## Abstract

The androgen receptor (AR) plays key roles in the development of prostate tissue and the development and progression of prostate cancer (PC). AR guides cytodifferentiation and homeostasis in benign luminal epithelial cells; however, in PC, AR instead drives the uncontrolled proliferation of these cells. This ‘AR malignancy shift’ (AMS) is a central event in tumorigenesis. Using a ChIP‐seq approach in primary human tissues, cell lines, and mouse models, we demonstrate that the AMS occurs in every sample analyzed, suggesting that it is necessary for PC development. Using molecular and genetic techniques, we demonstrate that forkhead box (FOX)A1, HOXB13, GATA2, and c‐JUN are involved in the regulation of the AMS. AR‐binding sites (ARBS) are enriched for FOX, HOX, and GATA motifs in PC cells but not for c‐JUN motifs in benign cells. We show that the SPOP mutation commonly found in localized PCs can cause the AMS but is not transformative on its own and must be coupled to another mutation to transform cells. We show that the AMS occurs in mouse models of PC as well and that chronic low T, which is associated with increased PC risk and aggressiveness in humans, also causes the AMS in mice. We have discovered a previously unrecognized, fundamental tenet of PC, one which explains how and why AR signaling is different in cancer and benign cells. Our work has the potential to be used to stratify patients with localized PC for specific treatments. Furthermore, our work suggests that the AMS is a novel target for the treatment and/or prevention of PC.

## Graphical abstract

The androgen receptor plays key roles in the development of prostate tissue; however, an ‘AR malignancy shift’ (AMS) can occur which allows AR to also drive prostate cancer (PC). We have characterized this shift, and our work has the potential to be used to stratify patients with localized PC for specific treatments and suggests that the AMS is a novel target for the treatment and/or prevention of PC.

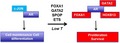

**Abbreviations**
AMS: androgen malignancy shift
AR: androgen receptor
IPA: ingenuity pathway analysis
N‐ARBs: normal androgen receptor‐binding sites
PC: prostate cancer
T: testosterone
T‐ARBs: tumor androgen receptor‐binding sites
TF: transcription factor
WT: wild‐type

## 1 Introduction

Despite advances in detection and treatment, prostate cancer (PC) remains the second leading cause of cancer death in American men (Siegel *et al.*, 2013). The prostate is a primary target organ of androgens, and androgen receptor (AR) signaling is required for prostate development (Litvinov *et al.*, 2003; Marker *et al.*, 2003). Following male development, AR guides cytodifferentiation and homeostasis in benign luminal epithelial cells (Isaacs, 1984; Kyprianou and Isaacs, 1988; Sugimura *et al.*, 1986). However, in PC, AR instead drives the uncontrolled proliferation of these cells; this change in AR behavior is a central event in tumorigenesis, as the AR becomes a primary driver of growth in malignant neoplastic cells (Copeland *et al.*, 2018). Descriptions of the difference in AR behavior in benign and PC cells go back nearly two decades to pioneering work from Gao and Isaacs, who demonstrated ‘that during transformation of androgen‐responsive normal prostatic epithelial to malignant cancer cells, a shift in the AR axis occurs’ (Gao *et al.*, 2001). However, there has never been a mechanistic description of this phenomenon, with little to no research into what causes this shift, what the shift looks like at a molecular level, or even what AR‐regulated genes define one state from the other.

Using unbiased genomic approaches, multiple groups have reported thousands of AR‐binding events to chromatin in PC cell lines and tissues, showing different binding patterns in different stages of cancer (Jin *et al.*, 2013; Massie *et al.*, 2011; Sharma *et al.*, 2013) . In one of the earliest such studies, Wang *et al.* (2007) used AR ChIP‐chip to identify dihydrotestosterone‐induced AR‐binding sites (ARBS) in LNCaP cells. The authors found a significant co‐occurrence of an AR half‐site motif with other transcription factor (TF)‐binding motifs including forkhead box (FOX) and GATA. AR’s association with these motifs in PC cells has been confirmed in other studies (Sharma *et al.*, 2013). Follow‐on studies have focused on the reprogramming of ARBS in metastatic PC cells during the transition from a hormone‐sensitive state to a castration‐resistant state and have revealed that many additional factors can influence this transition (Mills, 2014). However, little is known of AR‐binding patterns in the benign prostate or early‐stage PCs. Pomerantz *et al.* (2015) recently reported AR ChIP‐seq data from a small series of paired benign/PC tissues. They identified ARBS that were unique to tumor tissue (T‐ARBS) or to benign/normal tissue (N‐ARBS) and demonstrated that these unique ARBSs could be used to segregate normal tissue samples from tumor tissue samples. This change in ARBS preference, what we call the AR malignancy shift (AMS), provides an important first molecular description of the phenomenon Gao and Isaacs described years ago. Pomerantz et al. further found that T‐ARBSs were enriched for FOX and HOX motifs compared to N‐ARBSs. They demonstrated in subsequent experiments that overexpression of FOXA1 and HOXB13, two TFs previously shown to be associated with PC progression (Brechka *et al.*, 2017; Yang and Yu, 2015), induced a change in the AR‐binding pattern in a benign prostate cell line such that it looked like that of PC cells (i.e., switching from predominantly N‐ARBSs to T‐ARBSs). From this excellent work, we now have a measurable ‘read‐out’ of the AMS at the molecular level; the change of AR binding from predominantly N‐ARBSs to T‐ARBSs wherein there is an increased association of ARBSs with FOX, HOX, and as we show here, GATA motifs, and a decreased association with JUN motifs.

In this report, we add to our knowledge of the AMS with additional AR ChIP‐seq and molecular experiments to show that the AMS occurs in ALL human samples studied to date and that GATA2 and c‐JUN are integral components of the AMS machinery in addition to FOXA1 and HOXB13. We also show that the drivers of the AMS are some of the alterations that are frequently found in localized PCs and that these alterations are not tumorigenic on their own but must be coupled to other mutations for prostate epithelial cell transformation. Finally, we show that the AMS can be modeled in mice, that it occurs in multiple genetic models of PC, and that low testosterone (T) can cause the AMS in benign mouse prostate tissue.

## 2 Materials and methods

### 2.1 Patient samples

De‐identified patient samples were received from City of Hopes’ umbrella discarded tissue protocol for pilot studies as approved by the City of Hope ethics committee. Eleven normal and three tumor (two matched) fresh tissue samples were taken at time of radical prostatectomy and immediately snap‐frozen in OCT and stored at −80° C. A 5‐µm section was used for H&E staining by the City of Hope pathology core and areas of tumor and benign identified by a pathologist. A precooled scalpel was used to isolate the tumor and normal cells on a bed of dry ice to keep samples frozen and then stored at −80 °C until used in subsequent experiments.

### 2.2 Mouse models

All animal studies were carried out in accordance with the recommendations in the Guide for the Care and Use of Laboratory Animals of the National Institutes of Health and were approved by the City of Hope IACUC #13023. Models of low T were created as previously described (Zhou *et al.*, 2017; Zhou *et al.*, 2013); briefly, 8‐week‐old male wt C57BL6 mice (Taconic, Rensselaer, NY, USA) were divided into four groups: intact, castrate, castrate + silastic capsule with a physiological low T release rate, and castrate + a capsule with a release rate of physiological normal T. Blood was routinely collected to monitor serum T levels. T levels in this model have been previously extensively characterized by ELISA and mass spectrophotometry (Zhou *et al.*, 2017; Zhou *et al.*, 2013). In this experiment, blood was collected by tail vein (3 weeks for 6‐week cohorts; 8 and 48 weeks for long‐term cohorts) and by terminal cardiac puncture upon euthanasia and ELISA determined the serum T concentration. Prostates were collected 6 weeks postsurgeries for short‐term studies (*n* = 5 for each cohort) and another after chronic low T study consisted of a normal cohort (*n* = 9) and a low T cohort (*n* = 7) had prostates collected at 60–80 weeks and immediately frozen down and stored at −80 °C. Mouse prostate tissue from the *Nkx3.1^+/−^; Pten^+/−^* model, and appropriate control tissue from mice of the same background was a kind gift from the Abate‐Shen Laboratory (Herbert Irving Comprehensive Cancer Center, New York, NY, USA).

### 2.3 Cell culture

The LHSAR cells were a kind gift from the Freedman laboratory (Pomerantz *et al.*, 2015) and were maintained in PREGM media (Lonza, Alpharetta, GA, USA). E8 cells were a gift from the Roy‐Burman laboratory (Liao *et al.*, 2010) and were maintained in Dulbecco’s modified Eagle’s medium with 10% FBS (Gibco, Waltham, MA, USA). LAPC4 cells were a gift from the Charles Sawyers laboratory (Klein *et al.*, 1997) and were maintained in RPMI with 10% FBS (Gibco). BPH1‐AR cells were created by stably transfecting human AR in BPH‐1 cells (Jones *et al.*, 2009) and were maintained in RPMI with 10% FBS (Gibco). All media were supplemented with 1% pen/strep (Corning, Corning, NY, USA) and cultured in uncoated filter top polystyrene flasks maintained at 37 °C in 5% CO_2_ in humidified air. Cells were split at 80% confluency using 0.25% trypsin in EDTA (Gibco).

### 2.4 Transfections

Cells at ~ 75% confluency were transfected using the Lipofectamine LTX™ kit with Plus reagents (Thermo Fisher, Waltham, MA, USA) as per standard protocol with various plasmids and siRNAs. Specifically, the plasmids were vector (pIRESHyg2; Takara, Kusatsu, Shiga Prefecture, Japan), FOXA1 (HsCD00455927; Harvard, Cambridge, MA, USA), FOXA1 L455M was created by site‐directed mutagenesis, GATA2 (HsCD00456004; Harvard), PIK3CA (PIK3CA E545K; Addgene, Cambridge, MA, USA), and SPOP (pDONR223_SPOP_p.F133S; Addgene). For transient GATA2 and c‐JUN siRNA transfections, the Flexitube™ siRNA kits were used (Qiagen, Hilden, Germany) as per standard protocol.

### 2.5 Cell colony‐forming assay

After 48 h, the cells were harvested and 20 000 cells were seeded in 96‐well plates in triplicate for each treatment for subsequent CytoSelect™ soft agar cell colony‐forming assays (Cell Biolabs, San Diego, CA, USA) to analyze cell transformation via attachment independent growth rates as per standard protocol. Statistical analysis was by a two‐way *t*‐test in prism V 7.02 (GraphPad, San Diego, CA, USA). A *P‐*value of > 0.05 was considered significant.

### 2.6 ChIP

ChIP was performed as previously described (Leung *et al.*, 2013). Briefly, between 150 and 300 mg of frozen tissue was cross‐linked in 100–200 µL of 1% formaldehyde and homogenized in the Stormbullet™ for 3 × 3 min on full power with 2–9 mm stainless beads (Next Advance, Rensselaer, NY, USA) and formaldehyde was quenched with 125 mm glycine for 5 min. Cells in 150 mm dishes were harvested at ~ 80% confluency and fixed in formalin for 10 min and quenched for 5 min at room temp before washing 2× in cold PBS and then kept on ice. 0.1% and 0.5% SDS buffers were used to isolate and then disrupt nuclei, respectively. Chromatin was sheared in the Bioruptor™ pico (Diagenode, Denville, NJ, USA) to 100–500 bp in length with six cycles (30 s on/30 s off). Lysates were precleared with Pierce A/G magnetic beads (Thermo Fisher), and 10% of lysate was stored at −80 °C as input. IP was overnight at 4 °C with anti‐AR antibody (8 µg of N‐20x; Santa Cruz, Dallas, TX, USA or 10 µg of 74272; Abcam, Cambridge, MA, USA) or 10 µg rabbit IgG. Antibody/chromatin conjugates were isolated with A/G magnetic beads. Chromatin was uncross‐linked overnight at 65° C with proteinase K and DNA extracted with phenol–chloroform. For quality control, 5 µL was run out on an agarose gel for chromatin size determination and DNA quantified and quality checked on a NanoDrop (for input; Invitrogen) or q‐bit dsDNA HS assay kit (for IP; Invitrogen).

Due to the discontinuation of the N20× anti‐AR antibody from Santa Cruz during this study, which had previously been used in the majority of AR ChIP‐seq published papers, we sought an alternative antibody and compared AR ChIP‐seq with remaining stocks of the N20× antibody and the 74272 ChIP grade antibody (Abcam). While there were some differences between the two, including more promiscuous binding by the Abcam antibody, the two antibodies performed similarly in our hands (Fig. S1, and discussed below).

### 2.7 Library preparation and sequencing

ChIP‐seq libraries were prepared with Kapa DNA HyperPrep Kit (Roche, Wagistrasse, Schlieren, Switzerland) according to the manufacturer's protocol. Briefly, 5–10 ng of immunoprecipitated DNA underwent end‐repaired, A tailing, and adaptor ligation. Ten cycles of PCR were performed to produce the final sequencing library. The libraries were validated with the Agilent Bioanalyzer DNA High‐Sensitivity DNA Kit (Agilent, Santa Clara, CA, USA) and quantified with Qubit (Invitrogen). ChIP‐seq libraries were sequenced on Illumina HiSeq 2500 with V4 SBS reagent in the single‐read mode of 51 cycles of read 1 and 7 cycles of index read. Real‐time analysis 2.2.38 software was used to process the image analysis and base calling.

### 2.8 Alignment and peak calling and QC

ChIP DNA from each sample was barcoded and sequenced on the HiSeq 2500 to generate 51‐bp single‐end reads. Sequenced reads were aligned to the human (hg19) or mouse (mm9) reference genome using NovoAlign (http://www.novocraft.com/products/novoalign/). Mapped reads were filtered to exclude PCR duplicates using Picard (https://broadinstitute.github.io/picard/). Data quality measurements and fragment length estimation were performed using Phantompeakqualtools (Kharchenko *et al.*, 2008; Landt *et al.*, 2012). Peaks were identified using MACS2 (Zhang *et al.*, 2008) with a *P*‐value threshold of 0.1, followed by a secondary filtering with *q*‐values of 0.1, as described previously (Datta *et al.*, 2018). Summaries of alignment reads and MACS2 peak calling are provided in Tables S1 and S2, respectively.

### 2.9 AR/DNA‐binding motif analysis (heatmaps)

A set of previously identified ARBS unique to tumor (T‐ARBs; *n* = 9179) or normal tissue (N‐ARBs; *n* = 2690) (Pomerantz *et al.*, 2015) was used to calculate RPB (reads per billion per nucleotide) within the T‐ARBs and N‐ARBs for each of our samples, as described previously (Datta *et al.*, 2018), with white pixels for zero and negative and red pixels for positive with a contrast value of 1.0. Unsupervised clustering was then performed using Java TreeView version 1.1.6r4 (Saldanha, 2004).

### 2.10 Motif enrichment analysis

The Galaxy/Cistrome ‘seqPos Motif tool’ was used to discover candidate TF motifs both in our AR ChIP‐seq data and that of publicly available datasets (Liu *et al.*, 2011). We then quantified the frequency of specific position weight matrices (PWMs) of selected TF motifs in the ARBSs from our and publicly available datasets. The specific human PWMs were AR (MC00465 and MC00468), GATA2 (MC00118), HOXB13 (MC00488), FOXA1 (MC00311), c‐JUN (MC00321), and CEPBα (MC00273). First, the genome‐wide occurrences of these motifs were identified using Homer (scanMotifGenomeWide.pl) with log odds detection threshold of 5 (Heinz *et al.*, 2010). The occurrences were then used to calculate the percentage of peaks in each containing specific motifs. Statistical analysis of motif enrichment between different samples (treatment groups or matched T/N cohorts) was done with a chi‐square test using R software environment (https://www.r-project.org/). A *P‐*value of > 0.05 was considered significant.

### 2.11 Motifs peak overlap regions

Galaxy/Cistrome (Liu *et al.*, 2011) was used to analyze overlap of identified peaks to create Venn diagrams.

### 2.12 Ingenuity pathway analysis (IPA)

Tumor and normal specific AR ChIP peaks were loaded into IPA (Qiagen) to identify genes canonical pathways, disease networks associated with the peaks.

### 2.13 Quantitative PCR

ChIP‐qPCR was used to quantify the enrichment of IP compared to input as previously described (Bolton *et al.*, 2007) including the primer sequences that were used. 2–4 µg of purified DNA was used as target with the Taq PCR core kit (Qiagen). Statistical analysis was by a two‐way *t*‐test in prism V 7.02 (GraphPad). A *P‐*value of > 0.05 was considered significant.

## 3 Results

### 3.1 The AR malignancy shift occurs in all human samples analyzed to date

**Figure 1:**
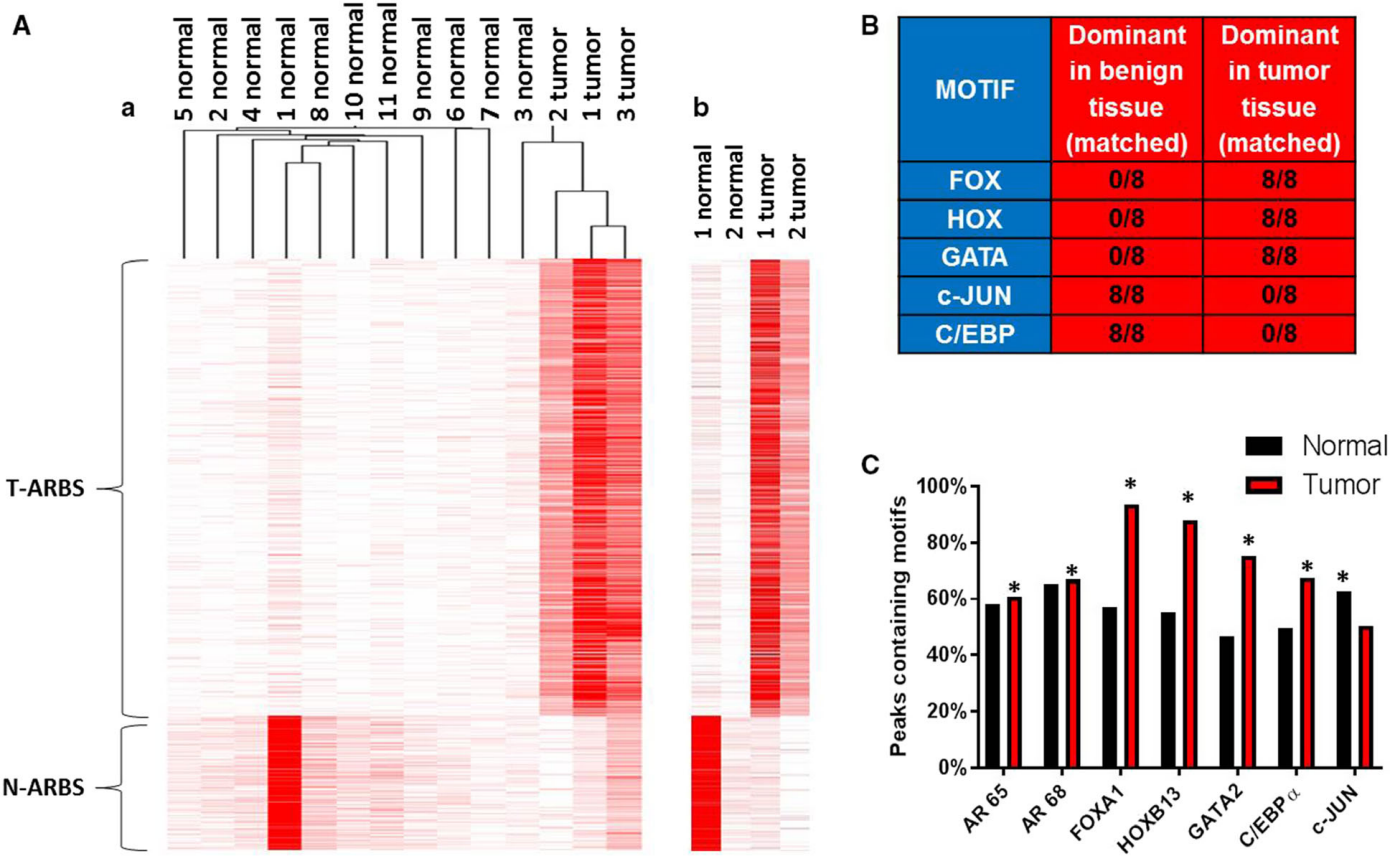
Discovering and defining components of the AMS. Using our bioinformatics methods, we reanalyzed the data from the Pomerantz samples along with our new samples. (A) Unsupervised hierarchical clustering of ARBS enrichment demonstrates a segregation of normal from tumor samples. A side‐by‐side comparison of our two matched samples is also shown. (B) Motif discovery was performed on all matched samples. Shown is a summary of indicated motifs in benign and tumor sections of the samples. (C) The percentage of motifs of specific TF family members were quantified in normal and tumor peaks. Statistical analysis of motif enrichment between groups was by chi‐square test using r software environment, and a *P‐*value of > 0.05 was considered significant as indicated by a *.

Previous work by Pomerantz *et al.* (2015). demonstrated that AR binds to unique sites in tumor and normal prostate tissue. We sought to confirm the utility of the Pomerantz N‐ARBSs and T‐ARBSs to segregate normal from cancer tissue in our own patient cohort. To validate our bioinformatics methods, which were very similar to those used in Pomerantz *et al*., we first reanalyzed the publicly available AR ChIP‐seq data from the Pomerantz *et al.* (2015) patient samples (normal = 7, tumor = 13; Gleason 3 + 3 to 5 + 5) and were able to recapitulate the robust segregation of cancer from normal samples via unsupervised hierarchical clustering (Fig. S2). We next performed the same analysis on an additional 14 patient samples (normal = 11, tumor = 3; Gleason 3 + 3 and 3 + 4) demonstrated clear segregation of normal from tumor (Fig. 1Aa). Two of our patients had matched tumor/normal tissue, and an isolated side‐by‐side analysis clearly shows the difference between tumor and normal tissue (Fig. 1Ab). We have thus shown that the N/T‐ARBSs defined in Pomerantz et al*.* can be used to delineate tumor from normal tissue in an independent patient cohort and, importantly, that the difference in AR‐binding patterns exists in every single patient sample analyzed to date.

Many AR‐regulated genes have been identified in PC cells and tissues, but little is known concerning genes that are regulated by AR selectively in benign versus tumor tissue. To begin to understand what affect the differences in AR binding have on benign and cancerous prostate epithelial cells, we identified AR peaks that were unique to benign and cancer tissue from our patients and created lists of putative ‘peak‐associated genes’. We then used those lists to perform gene ontology analyses using IPA. In normal tissue, ‘germ cell–Sertoli cell junction signaling’, ‘ERK/MAPK signaling’, and ‘HIPPO signaling’ were the top hits, while in tumor tissue, ‘IGF‐1 signaling’, ‘ephrin A signaling’, and ‘androgen signaling’ were among the top hits. Our results were very similar to gene ontology analyses that were performed in Pomerantz *et al*. The analyses of normal gene sets share 5 of the top 20 ‘upstream regulators’, and the analyses of tumor gene sets share 10 of the top 25 ‘upstream regulators’. We also found genes associated with unique normal and tumor AR peaks (Tables S3 and S4). This information could be used to create additional biomarkers to distinguish tumor from normal prostate tissue and to better understand the biological differences between the normal and cancerous states.

### 3.2 FOXA1, HOXB13, GATA2, and c‐JUN are integral components of the AMS

To gain a better understanding of the regulation of the AMS, we performed TF motif analysis of ARBSs in normal tissue and tumor tissue. Pomerantz *et al*. previously demonstrated an enrichment of FOXA1 and HOXB13 motifs in T‐ARBS compared to N‐ARBS. For their analysis, they combined AR ChIP‐seq peaks from all tumor samples and compared them to all benign samples. Interestingly, a superficial reanalysis of the individual patient samples from the Pomerantz cohort shows that in every case, FOX and HOX motifs are more enriched in the AR peaks from cancer specimens than in the matched normal samples (Fig. 1B). We also observed that other motifs were consistently enriched in either the normal or cancer tissue, including GATA in cancer, and JUN and CEBP family members in normal. We therefore quantified the enrichment of motifs for specific members of each of the TF families represented by the general motifs. FOXA1, HOXB13, GATA2, CEBPα, and c‐JUN were chosen due to the extensive literature supporting the role for each in human PC and AR regulation (Hsu and Hu, 2013; Pomerantz *et al.*, 2015; Wang and Koul, 2017; Wu *et al.*, 2014). Looking specifically at the 10 matched patient sample datasets (eight from Pomerantz + two from our cohort), we found that the AR motif itself was not significantly different between tumor and normal datasets, but FOXA1 (*P‐*value =< 0.001), HOXB13 (*P‐*value =< 0.001), and GATA2 (*P‐*value =< 0.001) motifs were significantly enriched in tumor compared with normal tissue, and conversely, c‐JUN was enriched in normal tissue (*P‐*value =< 0.001) (Fig. 1C). CEBPα did not match our initial analysis so we did not investigate this TF further.

**Figure 2:**
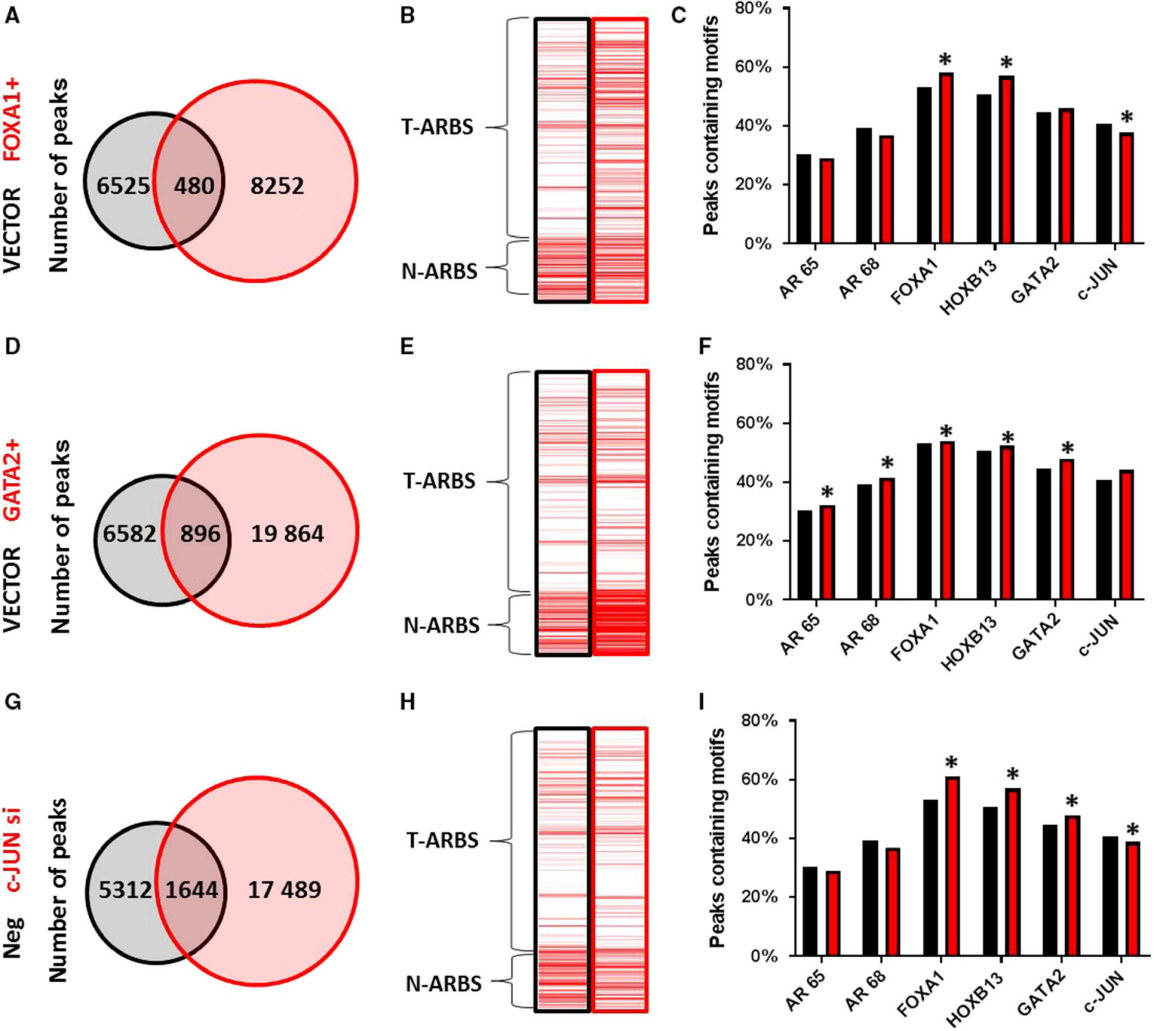
Defining TFs that are functional components of the AMS. The benign LHSAR prostate cells were transfected with FOXA1 or GATA2 expression plasmids or c‐JUN siRNAs, or appropriate controls, and AR ChIP‐seq was performed 2 days later. Venn diagrams of shared and unique peaks compared to controls (A, D, and G) demonstrate an expansion of ARBSs in each. Enrichment for Pomerantz T‐ARBSs and N‐ARBSs was performed for each experimental and control sample (B, E, and H) and shows different effects on AR binding. The percentage of TF motifs was determined for each sample (C, F, and I), again showing different effects on motif occurrence. Statistical analysis of motif enrichment between groups was by chi‐square test using r software environment, and a *P‐*value of > 0.05 was considered significant as indicated by a *.

Pomerantz *et al.* went on to demonstrate that overexpression of FOXA1 and HOXB13 in LHSAR cells, a benign human prostate epithelial cell line, changed the AR‐binding patterns such that they went from being significantly enriched in N‐ARBSs to being significantly enriched in T‐ARBSs (i.e., they went from looking like normal tissue to PC tissue). Conversely, knockdown of FOXA1 in LNCaP PC cells caused them to adopt AR‐binding patterns more similar to benign cells. We repeated these experiments in LHSAR cells by overexpressing FOXA1 or GATA2, or knocking down c‐JUN (Fig. S2A). Similar to what Pomerantz *et al*. observed, transfection with FOXA1 resulted in an overall increase in the number of AR peaks (Fig. 2A), including a selective increase in T‐ARBSs (Fig. 2B). Changes in AR binding to representative AR peaks were confirmed by AR ChIP‐qPCR, as were changes in the transcript levels of peak‐associated genes for this and subsequent transfections (Fig. S3D–F). Motif quantification identified significant enrichment of FOXA1, HOXB13, and GATA2 motifs (*P *value =< 0.001, 0.01, and < 0.001, respectively) and also a decrease in c‐JUN motifs (*P* = 0.001) in AR peaks from FOXA1‐transfected cells compared to AR peaks from control cells (Fig. 2C). There was also a reduction in the percentage of canonical AR motifs as FOXA1 increased the number of noncanonical ARBS, confirming previous findings (Jin *et al.*, 2014; Robinson *et al.*, 2014; Sahu *et al.*, 2011).

Overexpression of GATA2 also resulted in an overall increase in the number of AR peaks (Fig. 2D), but in this case, it caused increases in both N‐ARBSs and T‐ARBSs (Fig. 2E). This was reflected in an enrichment of all of the relevant TF motifs in AR peaks from GATA2‐transfected cells (Fig. 2F). This suggests that GATA2 has a slightly different function in AR reprogramming than FOXA1. Knockdown of c‐JUN in LHSAR cells also led to an increase in the total number of AR peaks compared to control siRNA (Fig. 2G). This experimental manipulation was similar to FOXA1 overexpression as it shifted binding to increase the T‐ARBS:N‐ARBS ratio (Fig. 2H) and caused enrichment of FOXA1, HOXB13, and GATA2 motifs but decreased enrichment of c‐JUN motifs (Fig. 2I) in c‐JUN siRNA‐transfected cells. Thus, our data strongly suggest that GATA2 and c‐JUN are TFs in addition to FOXA1 and HOXB13 that associate with ARBSs and regulate AR binding selectively in normal and cancer cells.

### 3.3 Alterations that frequently occur in the localized PC genome can cause the AMS

**Figure 3:**
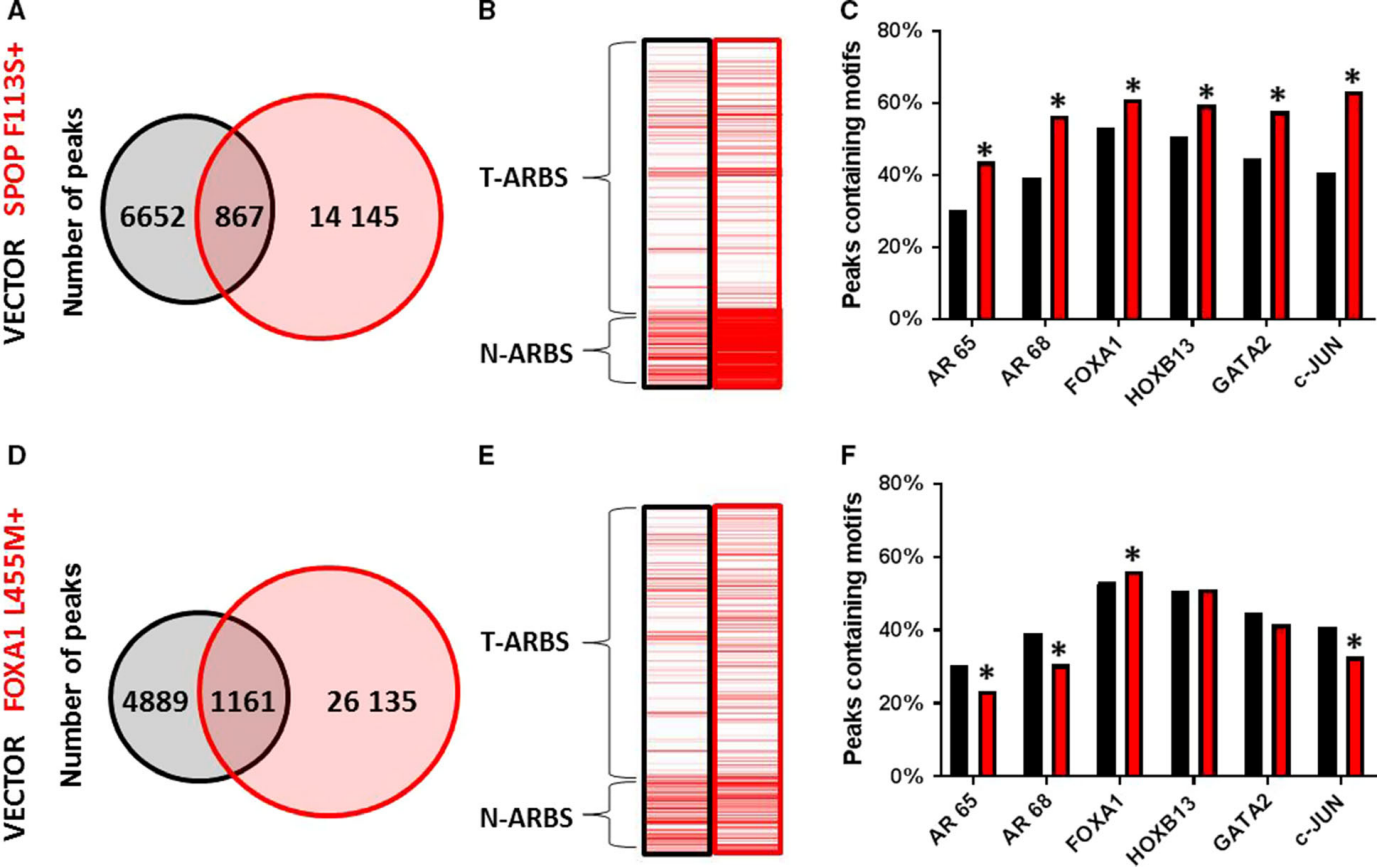
Recurrent mutations alter the AR cistrome. The benign LHSAR prostate cells were transfected with SPOP F113S or FOX A1 L445M expression plasmids or controls, and AR ChIP‐seq was performed 2 days later. Venn diagrams of shared and unique peaks compared to controls (A, D) demonstrate an expansion of ARBSs in each. Enrichment for Pomerantz T‐ARBSs and N‐ARBSs was performed for each experimental and control sample (B, E) and shows increased enrichment of T‐ARBSs. The percentage of TF motifs was determined for each sample (C, F) and shows different effects on motif occurrence. Statistical analysis of motif enrichment between groups was by chi‐square test using r software environment, and a *P‐*value of > 0.05 was considered significant as indicated by a *.

Many of the alterations that have been frequently identified in the genomes of localized, hormone‐sensitive PCs are known to occur in genes that encode for proteins that interact with AR or otherwise impinge upon the AR signaling axis (Copeland *et al.*, 2018). We investigated the ability of several of these proteins to alter AR‐binding patterns in LHSAR cells. *SPOP* mutations occur early in the natural history of PC, usually as heterozygous missense mutations with dominant‐negative and selective loss of function toward the remaining WT allele (Baca *et al.*, 2013; Boysen *et al.*, 2015). The SPOP protein is an E3 ubiquitin ligase adaptor that controls the ubiquitin‐mediated degradation of AR among other proteins, and its regulation of AR levels has been shown to be the key mediator of SPOP effects in PC (Geng *et al.*, 2014). We transfected SPOP F133S into LHSAR cells and found an increase in the total number of ARBS compared to control vector transfection (Fig. 3A). This was accompanied by increases of both N‐ARBSs and T‐ARBSs (Fig. 3B) and enrichments of all TF motifs in the AR peaks (Fig. 3C), similar to GATA2 overexpression. We also examined the effects of FOXA1 L455M, a missense mutation in the C‐terminal transactivating domain which has been identified in localized PC (Grasso *et al.*, 2012). Transfection of FOXA1 L455M had similar effects as overexpression of WT FOXA1, with an overall increase in the number of AR peaks (Fig. 3D), increased T:N‐ARBS ratio (Fig. 3E), and increased enrichment of FOXA1 and HOXB13 motifs with decreased enrichment of c‐JUN motifs (Fig. 3F). However, there were some slight differences compared to WT FOXA1 overexpression that suggest potential different effects, including no enrichment of GATA2 motifs, greater decrease in canonical AR motifs, and less increase in T‐ARBSs. Our data suggest that individual SPOP and FOXA1 mutations can alter AR binding in benign prostate cells such that the AR cistrome more closely resembles that of PC tissue than normal tissue.

### 3.4 AMS‐causing mutations must be coupled with another mutation to transform cells

**Figure 4:**
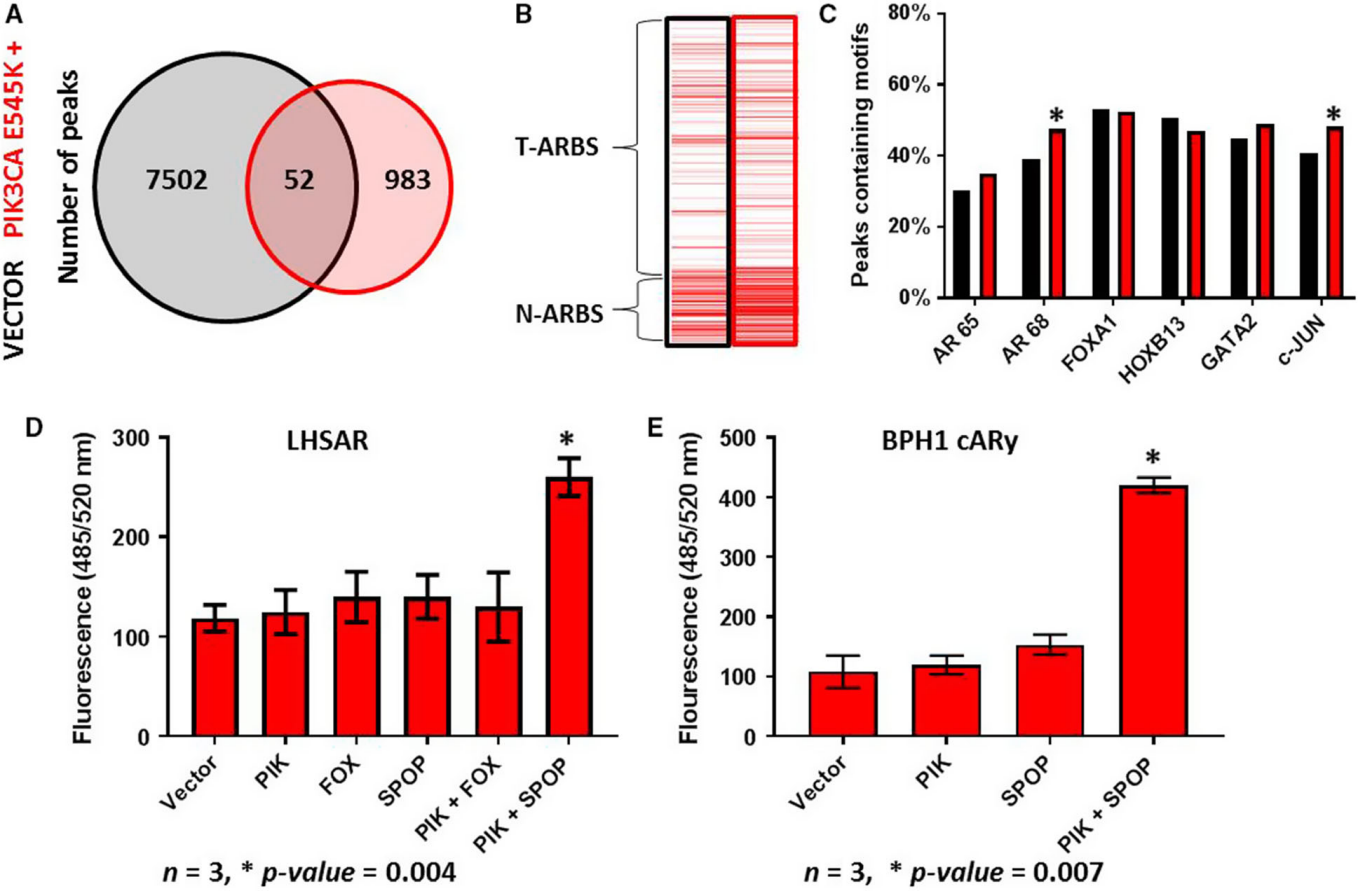
Mutations that cause the AMS are insufficient to transform cells on their own. The benign LHSAR prostate cells were transfected with a PIK3CA E545K expression plasmid or control, and AR ChIP‐seq was performed 2 days later. A Venn diagram of shared and unique peaks compared to controls (A) demonstrates a reduction of ARBSs. Enrichment for Pomerantz T‐ARBSs and N‐ARBSs was performed (B) and shows increased enrichment of N‐ARBSs. The percentage of TF motifs was determined (C) and suggests that PIK3CA mutant did not induce the AMS. (D) The indicated expression plasmids were transfected into LHSAR cells alone or in combinations, and a soft agar colony‐forming assay was performed. Growth was only observed with the combination of the PIK3CA and SPOP mutations. (E) The experimental results were recapitulated in the benign BPH1‐AR cells, which express ectopic AR. Statistical analysis of motif enrichment between groups was by chi‐square test using r software environment; a two‐way *t*‐test was used for analysis of the colony‐forming assay; a *P‐*value of > 0.05 was considered significant as indicated by a *; and error bars are SEM where *n* = 3.

The transformative potential of several of the mutations frequently found in the localized PC genome has been analyzed and few, if any, have been found to be able to transform cells or lead to the development of PC in mouse models on their own (Lehman and Stairs, 2015). We hypothesized that prostate cells require multiple alterations to become transformed, including one that causes the AMS. To test this hypothesis, we transfected SPOP F133S or FOXA1 L455M alone or in combination with PIK3CA E545K into benign LHSAR cells and quantified the anchorage‐independent growth in a colony‐forming assay. PIK3CA is frequently mutated or amplified in human PC, but has no known direct interaction or regulation of AR (Pearson *et al.*, 2018). Overexpression of PIK3CA E545K alone did not increase the number of AR peaks or result in increased T‐ARBSs, or alter TF motif associations in any way to indicate that the AMS had taken place (Fig. 4A–C). If anything, expression of this mutant in LHSAR cells made them look more like normal tissue, with increased N‐ARBSs and increased enrichment of c‐JUN motifs. Transfection of SPOP F133S, FOXA1 L455M, or PIK3CA E545K alone did not cause an increase in the ability of LHSAR cells to grow in soft agar in the colony‐forming assay (Fig. 4D). However, the combination of SPOP F133S with PIK3CA E545K significantly increased the growth of the cells, suggesting that they were transformed. This combination, but not either mutant alone, was also able to increase the anchorage‐independent growth of BPH‐1 AR cells, another benign prostate epithelial cell line (Fig. 4E). These data, combined with data from published mouse studies (discussed in the next section), support the idea that multiple mutations, including one that causes the AMS, are necessary for the transformation of prostate epithelial cells.

### 3.5 The AMS occurs in mouse models of prostate cancer

**Figure 5:**
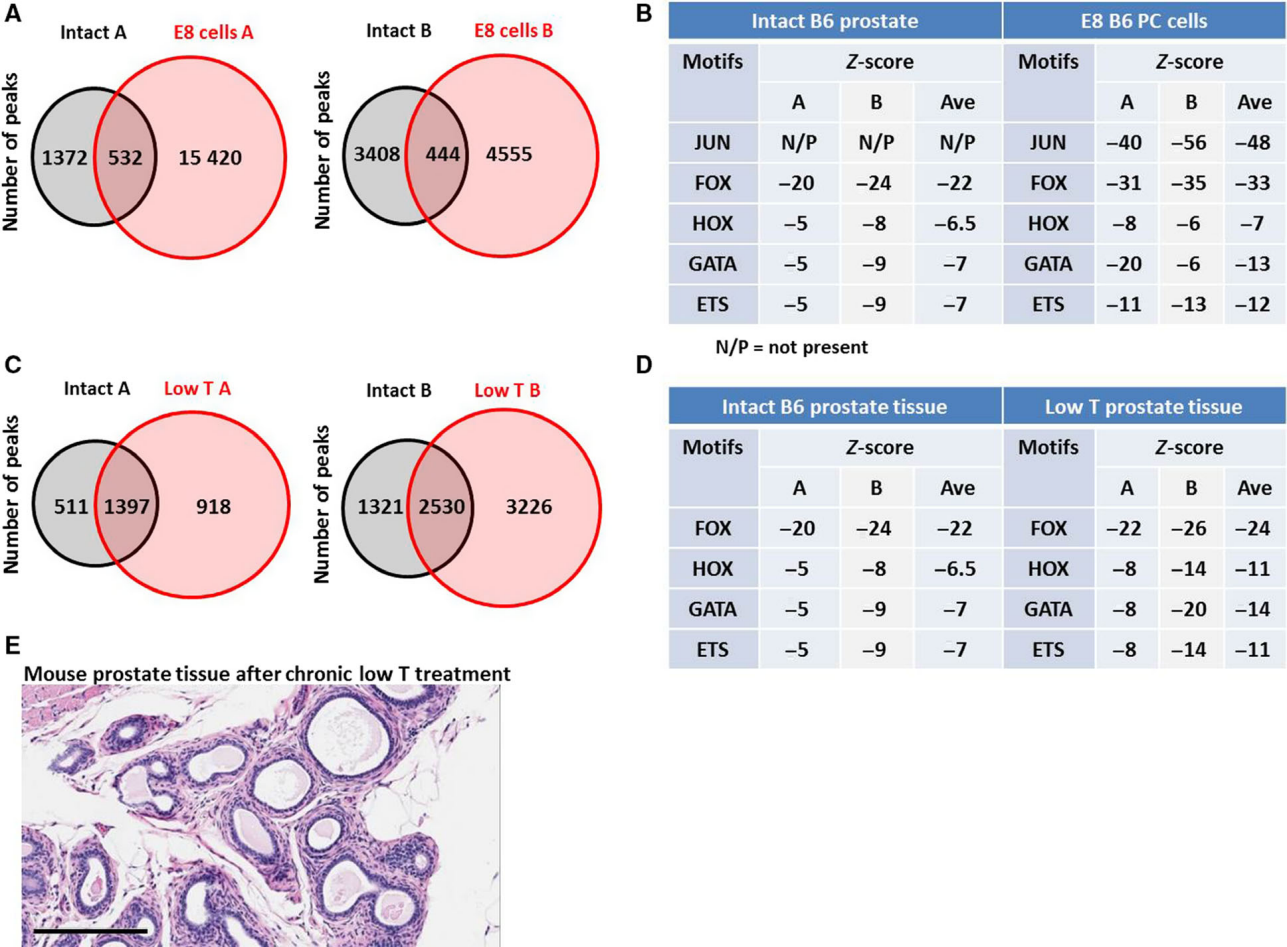
The AMS is present in *in vitro* and *in vivo* mouse models*.* AR ChIP‐seq was performed on E8 cells, which were derived from a hormone‐sensitive tumor from a *Pten^−^*/*^−^* mouse from a B6 background, on prostate tissue from healthy B6 mice, or on prostate tissue from B6 mice treated for 6 weeks with low T. Venn diagrams of shared and unique peaks compared to healthy prostate controls (A, C) demonstrate an expansion of ARBSs in each duplicate from both comparisons. Cistrome was used to determine the enrichment of TF family motifs in each sample, where a more negative z score indicates greater enrichment (B, D). (E) B6 mice were treated with low T for 18 months (*n* = 9); then, prostates were removed and examined histologically. No adenocarcinomas were detected in any mouse. Scale bar = 200 µm.

Our analyses suggest that the AMS occurs in all human PCs. We wanted to determine whether the AMS could be modeled in the mouse prostate. We performed AR ChIP‐seq on normal mouse prostate tissue as well as PC tissue from the *Nkx3.1^+/−^; Pten^+/−^* mouse model (Banach‐Petrosky *et al.*, 2007) and E8 cells, which were isolated from a hormone‐sensitive local prostate tumor from conditional *Pten^−^* ^/^ *^−^* mice (Liao *et al.*, 2010). In the E8 cells, we found an increased number of AR peaks compared to normal mouse prostate tissue from the same background (Fig. 5A,B). As we are unable to compare mouse to human AR peaks, we could not determine whether there was an enrichment of T‐ARBS or N‐ARBSs in these samples. We instead focused on identifying the TFs colocalizing with ARBSs in normal tissue and tumor tissue. Cistrome motif discovery found that AR, FOX, HOX, and GATA family motifs were significantly enriched in E8 cells compared to normal prostate tissue (Fig. 5C). Interestingly, we did not observe enrichment in JUN or CEBP family motifs in the normal tissue, as we did in human tissue. Unfortunately, the ChIP‐seq from the *Nkx3.1^+/−^;Pten^+/−^* mice failed our ChIP‐seq QC parameters, so we cannot report with any significance our findings from these studies, although Cistrome motif discovery did again suggest an enrichment of AR, FOX, HOX, and GATA family motifs.

Work from others also suggests that the AMS occurs in mouse models of PC. Chen *et al.* (2013)*.* created a mouse model in which a truncated ERG protein, like that seen with the TMPRSS2‐ERG translocation that occurs with great frequency in PC, was overexpressed selectively in mouse prostate epithelial cells. They found that ERG expression caused a > 4‐fold increase in the number of AR peaks compared to WT prostates, and their motif discovery found an increase in the percentage of GATA and AR family motifs in AR peaks from the ERG mice. ERG expression alone was not tumorigenic, but it did significantly accelerate adenocarcinoma development when coupled with *Pten* loss. This again supports the idea that mutations that cause AR cistrome changes are necessary, but not sufficient, for prostate tumorigenesis.

Our laboratory has a long‐standing interest in the association between low T in men and increased risk and aggressiveness of PC (Zhou *et al.*, 2013). We have shown that continued low T exposure in mice causes increased AR levels, increased local androgen synthesis, and changes in the transcriptome reminiscent of PCs in otherwise normal mouse prostate tissue (Zhou *et al.*, 2017; Zhou *et al.*, 2013). We decided to examine the effects of chronic low T on the AR cistrome. We found that in two independent experiments, low T treatment for 6 weeks increased the number of AR peaks compared to untreated mice (Fig. 5D,E). Cistrome motif analysis also found that FOX, HOX, and especially GATA family motifs were enriched in AR peaks from low T‐treated mice (Fig. 5D), indicative of the AMS. To determine whether low T alone can cause PC, we exposed seven mice with low T alongside nine intact mice and examined the prostates. Even after administration of low T for 20 months, there was no development of adenocarcinoma or histopathologic differences from WT mice (Fig. 5E). This suggests that, although low T can alter the AR cistrome in a manner consistent with the AMS, low T alone is not tumorigenic. Interestingly, when *Nkx3.1^+/−^;Pten^+/−^* mice were treated with low T, they developed PCs more rapidly than control animals (Banach‐Petrosky *et al.*, 2007). Exactly how low T causes the AMS is not yet known, but it does support the idea that the AMS must be coupled with an additional mutation for full transformation of prostate epithelial cells.

## 4 Discussion

Pomerantz *et al*. made an incredibly important contribution to our understanding of the role of AR signaling in PC initiation. The authors presented the first in‐depth analysis of AR‐binding patterns in matched benign and localized PC tissues. Their data laid the groundwork to allow us to better understand how AR changes from driving differentiation in benign cells to driving proliferation in cancerous cells. They provided a molecular underpinning of this behavioral change by identifying unique tumor and normal ARBSs, and further distinguished tumor from normal tissue by the frequency of FOX and HOX motifs near those ARBSs. These sites and TF motifs can be used to define the AMS. AR ChIP‐seq results from our patient samples confirm the utility of this operational definition of the AMS, as using their T‐ARBSs and N‐ARBSs clearly segregated our samples into normal or tumor by unsupervised hierarchical clustering. Certainly, the heatmaps derived from our samples are not as robust as those from Pomerantz *et al*. This is to be expected for several reasons. First, Pomerantz *et al.* derived the T‐ARBSs and N‐ARBSs from the same datasets that they then built the heatmaps from, so most of those peaks exist in all of their samples. Our data should not be expected to have all of the same peak calls. Furthermore, we used a different antibody for IP, as the manufacturer discontinued production of the antibody used in Pomerantz *et al.* Our comparison of antibodies (Fig. S1) shows a high concordance of AR peaks identified from the same specimens, but it is not identical. This, too, may have contributed to the less robust heatmaps. However, the similarity of our findings to those of Pomerantz *et al.* strongly suggests that the Abcam antibody can be used in place of the Santa Cruz antibody for future AR ChIP‐seq studies.

Using the functional definition of the AMS, the AR cistrome was found to be normal‐like in all 18 normal tissues and tumor‐like in all 16 tumor tissues. While many more samples will have to be sequenced, especially those from different genetic backgrounds, it is possible that the AMS is a universal phenomenon, one which is necessary for transformation of prostate epithelial cells.

**Figure 6:**
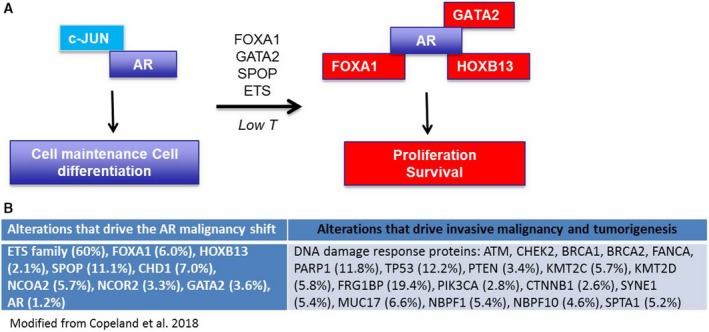
Model system of the AMS. (A) In benign cells, AR is associated with c‐JUN and drives a transcriptome resulting in homeostasis. Various factors and mutations are able to shift the AR cistrome so that AR is associated with GATA2, FOXA1, and HOXB13 and drives a transcriptome resulting in proliferation. (B) Mutations (and their frequency in the TCGA dataset) that potentially cause the AMS and those that potentially provide a second hit necessary for transformation.

Other important questions remain to be answered, including how, mechanistically, does AR change its binding sites and what can cause these changes? Identification of TF motifs associated with ARBSs provides some clues. Pomerantz *et al*. clearly demonstrated that FOXA1 and HOXB13 motifs were enriched in ARBSs in tumor tissue, and our results confirmed this finding. Our data and reanalysis of the Pomerantz data also demonstrated an increase in GATA2 motifs and a decrease in c‐JUN motifs in ARBSs in tumor tissue. Not only do these associated motifs provide additional benchmarks to discern the AMS, but they suggest that the TFs that bind to the motifs also play a role in differential AR binding. Initial motif identification experiments revealed motif families as enriched in tumor or normal tissue (e.g., FOX, HOX, GATA, JUN/AP‐1) with several specific members from each family listed as potential associated factors. FOXA1 and HOXB13 were chosen as specific family members to investigate because of their strong literature connections to AR signaling in PC, which is nicely described in Pomerantz *et al. *(2015). We chose GATA2 and c‐JUN as specific family members to investigate for the same reasons. GATA2 is a pioneering TF for AR and has been shown to change AR binding in PC, and expression of GATA2 is positively correlated to clinical and pathological outcomes in PC (Rodriguez‐Bravo *et al.*, 2017). The role of c‐JUN in PC is more controversial but decreased c‐JUN expression has been correlated to worse PC clinicopathological outcomes (Edwards *et al.*, 2004; Tamura *et al.*, 2007), and c‐JUN has been shown to interact with AR and enhance DNA binding (Bubulya *et al.*, 2001; Bubulya *et al.*, 2000). Our data and that from Pomerantz *et al*. clearly demonstrate that these four TFs influence the normal/tumor‐specific binding pattern of AR in human prostate epithelial cells. These findings suggest a model in which AR colocalizes to a greater extent with c‐JUN in normal tissue and with FOXA1, GATA2, and HOXB13 in tumor tissue (Fig. 6A). It should be noted that while c‐JUN acts on AR in luminal cells, it has increased expression in stromal cells where it regulates the AR cistrome (Leach and Buchanan, 2017; Leach *et al.*, 2017) so the reduced stromal:luminal cell ratio that occurs in PC could contribute to the decrease in c‐JUN motifs seen in AR ChIP‐seq data from the tumor samples. It is also possible that other FOX, HOX, GATA, and JUN/AP‐1 family members could be associated with tumor/normal ARBSs and could influence differential AR binding. Thus, other TF family members should be tested for their role in the AMS in future experiments.

Clearly not all of these TFs affected AR binding in the exact same way. The heatmap analysis of HOXB13 and FOXA1 overexpression in LHSAR cells in Pomerantz *et al*. demonstrates some differences in which ARBSs were occupied, and certainly, knockdown experiments demonstrated that HOXB13 affected AR levels but FOXA1 knockdown did not. Likewise, overexpression in this study of FOXA1 or GATA2, and knockdown of c‐JUN in LSHAR cells all had slightly different effects on AR binding. Although all three increased the total number of ARBSs, there were noticeable differences in the heatmaps and motif enrichments in ARBSs. For instance, FOXA1 overexpression increased the percentage of FOXA1, HOXB13, and GATA2 motifs and decreased the percentage of c‐JUN motifs, exactly correlating with the benchmarks of the AMS. GATA2 overexpression, however, increased the percentage of all motifs, including c‐JUN, and increased binding to both T‐ARBSs and N‐ARBSs. GATA2 has been shown to be a strong pioneer factor for AR (Rodriguez‐Bravo *et al.*, 2017), so it is perhaps not surprising to see that it increased binding to all types of sites. Although FOXA1 is also an AR pioneer factor, it appears to be more selective in which sites it increases access to and has been shown to act upstream of GATA2 (Zhao *et al.*, 2016). It is also important to consider the effects on AR levels in each of these experiments, as changes in AR levels themselves can affect the AR cistrome (Urbanucci *et al.*, 2011). As Pomerantz observed that HOXB13 levels influenced the levels of AR in LHSAR cells, we too examined the effect of our experimental manipulations on AR levels. We found that c‐JUN knockdown and FOXA1 overexpression did not significantly affect AR transcript levels, but GATA2 overexpression caused a significant increase in AR levels (Fig. S3D). GATA2 overexpression has been previously reported to increase AR mRNA and proteins levels (Böhm *et al.*, 2009) and GATA2 siRNA to reduce AR levels (Wu *et al.*, 2014), so our GATA2 data are consistent with the literature. It is possible that the increased AR levels could contribute to the expansion of ARBSs observed with GATA2 overexpression, as increased AR expression has been shown to positively correlate with number of ARBS and with AR‐binding affinity (Urbanucci *et al.*, 2011). In all, these data demonstrate that each factor regulates AR binding in different ways and that more work is needed to understand exactly how they coordinately control AR binding in a disease state‐selective manner.

It is also unclear what genetic alterations or other perturbations cause the AMS. As shown from Pomerantz’s and our data, simple changes in levels of FOXA1, GATA2, HOXB13, and c‐JUN can change AR binding in ways that are similar to the AMS. While the levels of these factors are known to change in some PC patients, and more work should be done to understand what controls the changes in their levels, this is unlikely to explain a significant fraction of the AMS. We hypothesized that many of the alterations frequently identified in the localized PC genome may be causes of the AMS (Fig. 6B). The most common alteration in localized PC from Caucasian men is increased expression of ETS family proteins, often via TMPRSS2‐ERG fusion (Tomlins *et al.*, 2005). Overexpression of ETS family members causes reprogramming of the AR cistrome and an altered transcriptional output that promotes invasion, autocrine signaling, and an aggressive tumor phenotype (Chen *et al.*, 2013). While motif analysis of ERG overexpression in mouse prostate tissue from Chen et al. shows no enrichment of FOX and HOX motifs, it does show enrichment for AR and GATA motifs compared to wild‐type (WT) mice (Chen *et al.*, 2013), suggestive of the AMS. Exactly how ERG overexpression causes changes in the AR cistrome is unclear. Interestingly, the percentage of AR and ERG peaks that physically colocalized in ERG mouse prostates was ~ 44%, which is highly significant, yet new AR peaks, those that did not exist in WT mice, had less overlap with ERG peaks than did the conserved AR peaks (~ 40% compared to ~ 60%) making it unlikely that ERG directly recruits the AR to new sites. However, a large fraction of new ARBS (77%) mapped to genes containing ERG sites (Chen *et al.*, 2013), raising the possibility of an ERG‐mediated field effect that promotes AR binding, perhaps by functioning as a pioneer factor. Several AR‐interacting factors have also been shown to function as pioneer factors, opening up chromatin to allow AR to bind to a wider array of sites, most notably, FOXA1 (Robinson *et al.*, 2014). FOXA1 is recurrently amplified and mutated in localized PC, and here, we show that FOXA1 overexpression and expression of FOXA1 L455M can alter the AR cistrome in a manner that is suggestive of the AMS. Pomerantz *et al*. suggested that FOXA1 acts as a pioneer factor to allow additional sites of binding for AR in tumor tissue, much like ERG is proposed to do in the Chen et al. study. However, it should be noted that both ETS and FOX family members can directly interact with AR (Chen *et al.*, 2013; Zhao *et al.*, 2016), so it is possible that the direct binding also influences AR‐binding preferences.

SPOP mutations, which are frequent in localized PC, also appear to cause the AMS. As SPOP functions in the E3 ubiquitin ligase complex, it is not likely to act as a pioneer factor, suggesting that it causes the AMS by a different mechanism. The *SPOP* mutations found in PC fail to recognize the AR; this decreases AR ubiquitination and degradation and leads to greater AR stability and AR‐mediated transcriptional output (Geng *et al.*, 2014). It has been shown in metastatic PC models that simple overexpression of AR causes changes in the AR cistrome (Urbanucci *et al.*, 2011), so it is possible that this is how SPOP mutation leads to the AR shift. Several other AR cofactors were found to be frequently mutated in the TCGA and other cohorts, including GATA2, NCOA2, and NCOR2 and CHD1 (Fig. 6B). These mutations likely affect the AR cistrome and deserve further investigation.

That multiple mechanisms converge on altering the AR cistrome in a similar fashion suggests a strong selective pressure of PC cells to do so, perhaps meaning that it is necessary for transformation and growth of nascent PCs. It also suggests why many attempts to classify localized PCs by common genomic alterations have failed to stratify patients by outcome. For instance, the TCGA study authors described ‘a molecular taxonomy in which 74% of the tumors fell into one of seven subtypes defined by specific gene fusions (ERG, ETV1/4, FLI1) or mutations (SPOP, FOXA1, IDH1)’ (Cancer Genome Atlas Research, 2015). While no study has been completed that assesses outcomes based on these exact classifications, an analysis using similar classifications failed to find differences in clinical outcomes among the groups (Tomlins *et al.*, 2015). This may be because, of the seven genotypes suggested by the TCGA analysis, six are defined by mutations which affect proteins that are strongly associated with AR reprogramming; several of these mutations have indeed been shown to cause the AMS, although they alone are unable to alone cause cancer in PC models. If each of the TCGA‐defined genotypes simply reflects a different path to the AMS rather than different mechanisms of tumorigenesis, then it may explain why the classification of tumors solely by TCGA‐defined genotypes has failed to reveal different clinical outcomes. Future experiments should attempt to identify all primary drivers of the AMS, and then differentiate tumors not by alterations that contribute to the AMS, but by those additional hits that are likely necessary to drive uncontrolled proliferation, invasive malignancy, and tumorigenesis (Fig. 6).

We provided additional *in vitro* evidence for the necessity of multiple hits to transform prostate epithelial cells. Neither SPOP F133S, which caused the AMS, nor PIK3CA E545K, which did not, were sufficient to allow growth of benign cells in soft agar in the colony‐forming assay, a surrogate for cell transformation. However, cotransfection significantly increased growth in two different benign cell lines. However, FOXA1 L455M, while causing AR cistrome changes, did not increase growth in this assay when cotransfected with PIK3CA E545K. The AR cistrome changes induced by this FOXA1 mutant were not as robust as those with SPOP, so it is possible that a full AMS had not occurred in these cells. It is also possible that different combinations of AMS‐causing and non‐AMS‐causing mutations are necessary for transformation of cells. However, studies from multiple groups suggest that this ‘two‐hit’ mechanism is necessary. Chen et al. demonstrated that ERG overexpression was not tumorigenic on its own, but accelerated PC kinetics when coupled with *Pten* loss (Chen *et al.*, 2013). Further support for this hypothesis comes from Blattner *et al.* (2017), where expression of SPOP F133V, a mutation very similar to the one we demonstrated caused changes in the AR cistrome (Fig. 3), did not by itself cause adenocarcinoma in the mouse prostate, but greatly accelerated the development of cancer when coupled with *Pten* loss. Chen *et al*. propose that in the case of ERG, ‘*ETS* factors cause prostate‐specific transformation by altering the AR cistrome, priming the prostate epithelium to respond to aberrant upstream signals such as *PTEN* loss’. We propose a similar theory where specific alterations can cause AMS and are mechanistically responsible for priming the cells for a 2nd driver hit that fully transforms the cells. These findings warrant a more thorough investigation of which combinations of frequent alterations are sufficient to transform benign prostate cells.

Importantly, we demonstrate that the AMS likely occurs in mouse models of PC, both in E8 cells from *Pten^−^*/*^−^* mice and likely in *Nkx3.1^+/−^; Pten^+/−^* mice, although this latter dataset did not pass our QC filters. Interestingly, there are no documented mutations other than *Pten* loss in E8 cells, and AR is WT. While it is possible that *Pten* loss causes the AMS, it is unlikely as *Pten* loss is thought to be a later event in PC and PTEN does not have a known direct connection to the AR protein (Lotan *et al.*, 2013; Morais *et al.*, 2015). It is more likely that the tumor from which the E8 cells were isolated acquired a mutation over time in addition to *Pten* loss, or even a mutation later *in vitro* as has been shown before to occur in LNCaP cells (Castanares *et al.*, 2016), one which caused the AMS and allowed for full transformation of the prostate epithelium. The *Pten^−^* ^/^ *^−^* model takes up to 12 months to develop PC, in most laboratories (Kwak *et al.*, 2013). Perhaps the delay in cancer development is due to the requirement for additional alterations to accumulate, including one which causes the AMS. It is possible that one cause of the AMS in these mice is declining serum T levels. Indeed, we demonstrated that low T causes the AMS but alone is not sufficient to cause cancer, even after long‐term exposure. Furthermore, the *Nkx3.1^+/−^; Pten^+/−^* mouse model coupled with low T displayed much more rapid and aggressive PC than the model with normal T levels (Banach‐Petrosky *et al.*, 2007). How low T causes the AMS is unknown, but we have previously shown that it can increase AR levels, as well as altering the immune microenvironment (Zhou *et al.*, 2017; Zhou *et al.*, 2013). The fact that low T causes the AMS, which we propose primes cells for transformation, could account for the correlations between low T and increased PC risk and PC aggressiveness (Copeland *et al.*, 2018).

## 5 Conclusion

In summary, we build on previous elegant work by, among others, Pomerantz *et al*. to better define the AMS and show that the AMS, while not sufficient to initiate PC alone, may be a universal requirement in all patients progressing to PC. While Pomerantz demonstrated that ARBSs are enriched for FOX and HOX motifs in tumor tissue, we further classify the AMS by demonstrating the enrichment of GATA motifs and decrease of JUN motifs in ARBSs from tumor tissue. We also show that the AMS is recapitulated not only in mouse models of PC but also in human and mouse cell culture models, thus facilitating further investigation of this phenomenon. Finally, we demonstrate that several common PC mutations can cause the AMS and suggest that this information may inform more accurate genetic classifiers for localized PC.

## Conflict of interest

The authors declare no conflict of interest.

## Author contributions

JJ conceived the project. BC and JJ designed the project, collected and analyzed data, and wrote the paper. JD analyzed data and SP helped conceive and design the project.

## Supporting information


**Fig. S1** **. **Comparison of the 74272 (Abcam) and N20× (Santa Cruz) anti‐AR antibodies.

**Fig. S2** **. **Recapitulation of previous heatmaps.

**Fig. S3** **. **Validation and analysis of transfections.

**Table S1** **. **Alignment summary.
**Table S2** **. **MACS2 peak calling summary.
**Table S3** **. **Genes associated with unique AR peaks in normal tissue.
**Table S4** **. **Genes associated with unique AR peaks in tumor tissue.
