# Supplementary figures and images for: Factors that influence the androgen receptor cistrome in benign and malignant prostate cells
Source: Mol Oncol. 2019 Oct 13;13(12):2616–32. doi: 10.1002/1878-0261.12572 (PMC6887583; doi:10.1002/1878-0261.12572)

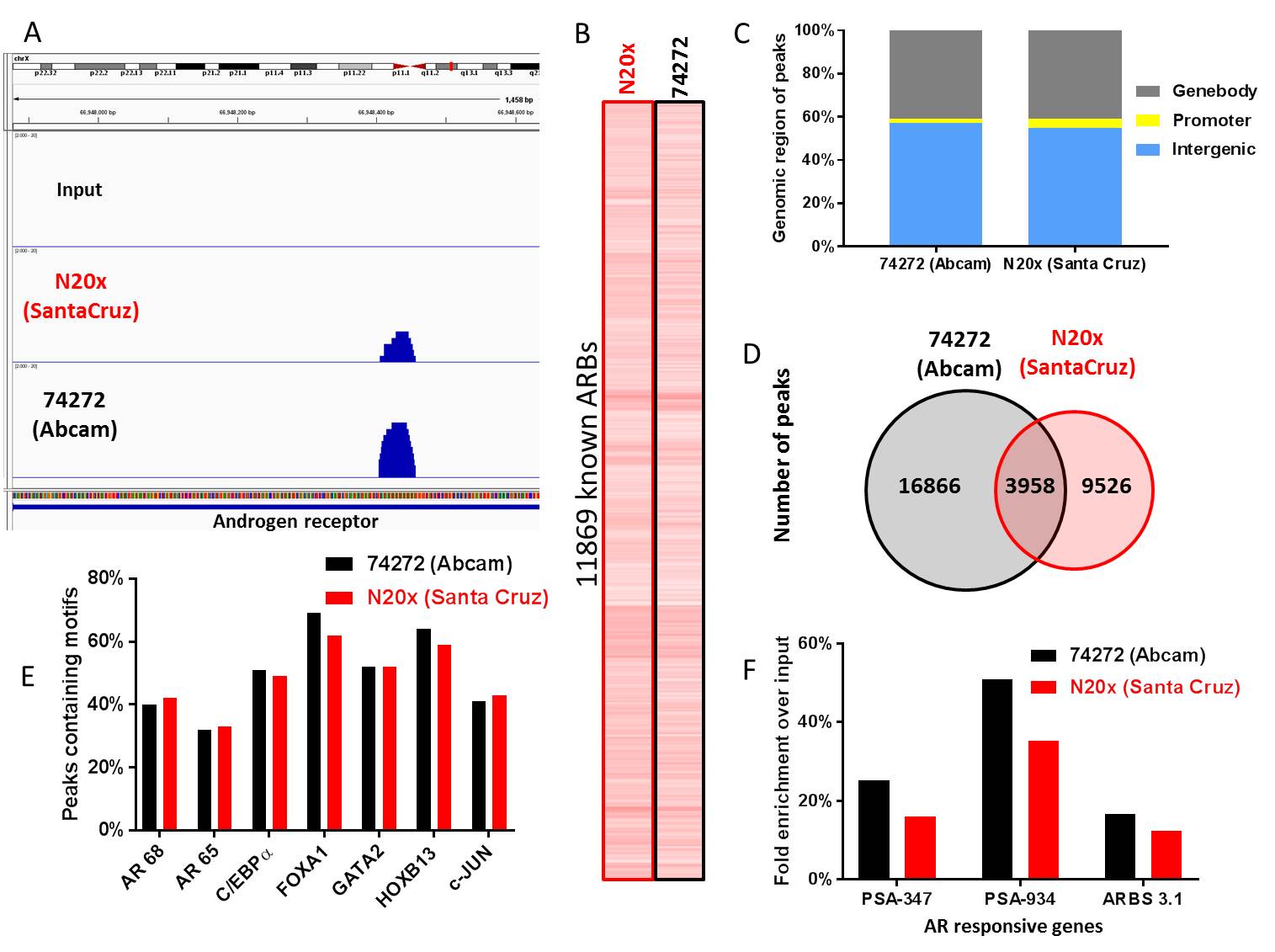

Supplement: Supplementary file 1 — Fig. S1 . Comparison of the 74272 (Abcam) and N20× (Santa Cruz) anti‐AR antibodies. [file MOL2-13-2616-s001.jpg]

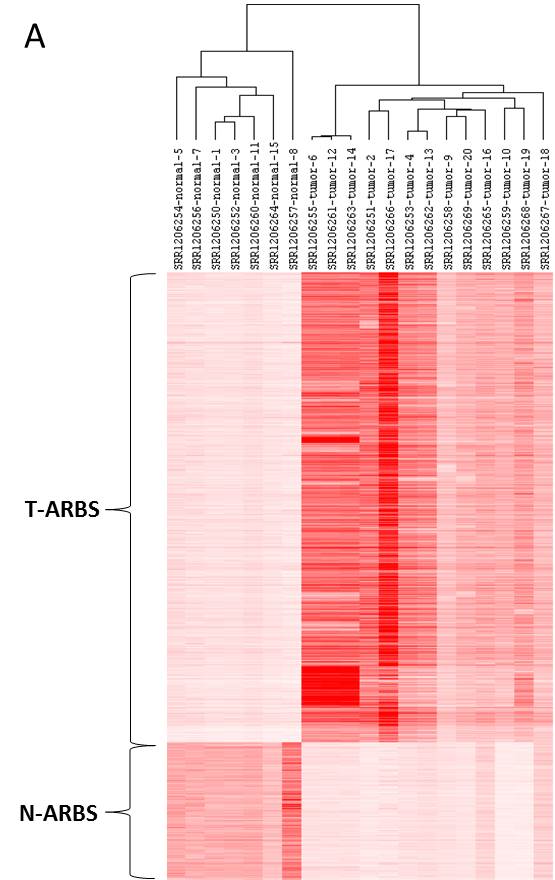

Supplement: Supplementary file 2 — Fig. S2 . Recapitulation of previous heatmaps. [file MOL2-13-2616-s002.jpg]

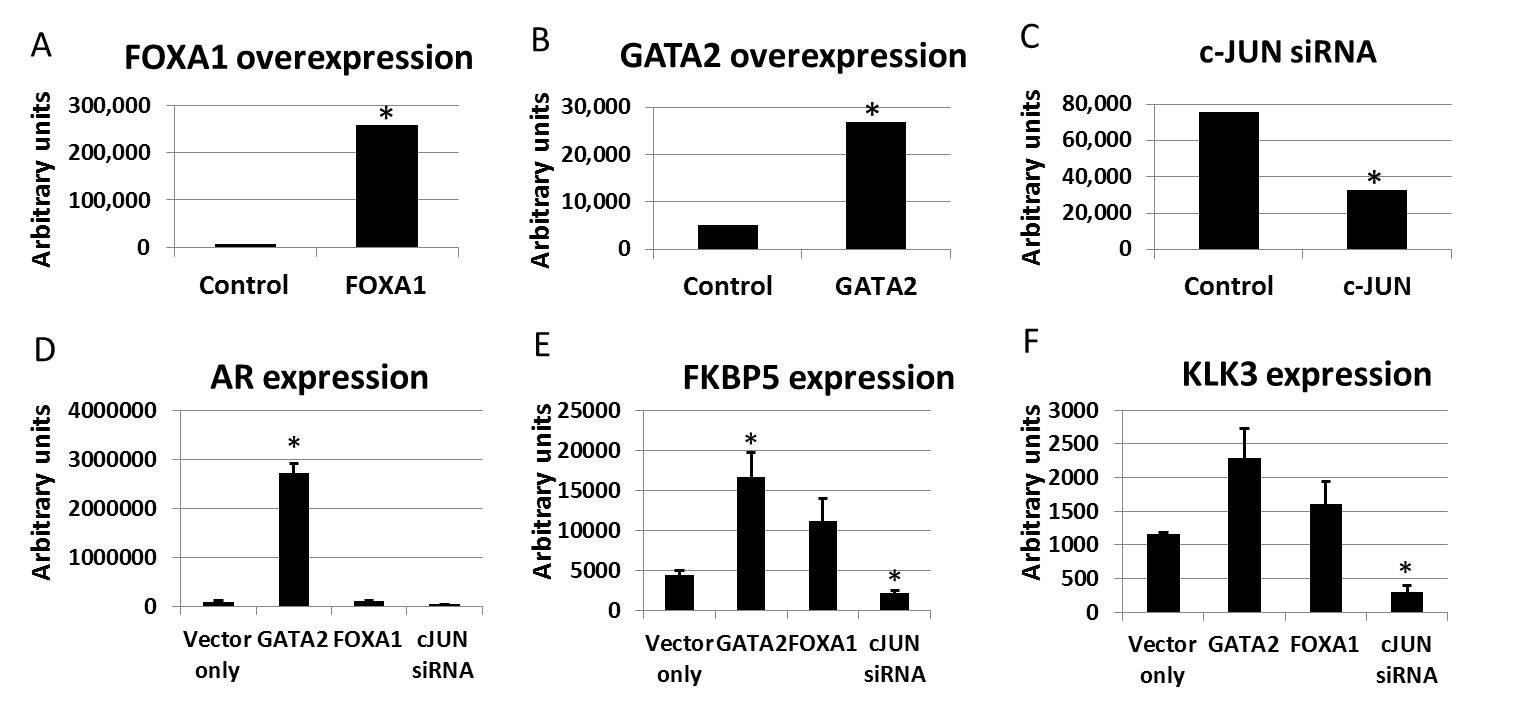

Supplement: Supplementary file 3 — Fig. S3 . Validation and analysis of transfections. [file MOL2-13-2616-s003.jpg]
