# Supplementary material for: Factors that influence the androgen receptor cistrome in benign and malignant prostate cells
Source: Mol Oncol. 2019 Oct 13;13(12):2616–32. doi: 10.1002/1878-0261.12572 (PMC6887583; doi:10.1002/1878-0261.12572)
Supplement: Supplementary file 5 [file MOL2-13-2616-s005.docx]

**Supplementary Figure 1. Comparison of the 74272 (Abcam) and N20x (SantaCruz) anti-AR antibodies.**

The equivalence of the two anti-AR antibodies was evaluated using LHSAR cells in parallel ChIP-seq experiments. (A) IGV tracks in the AR region were specific for both antibodies, albeit higher in Abcam with no enrichment in the input. (B) Similar heat-maps were produced from aligning peaks with known T-ARBS and N-ARBS AR peaks. (C) The genomic region annotations were similar between the two antibodies. (D) While the Abcam antibody resulted in more peaks with an overlap of ~27%, (E) the specific motif enrichments in the peaks were comparable (*p-value* > 0.001, E). (F) ChIP-qPCR of known ARBSs in the KLK3 gene showed comparable patterns of enrichment over input (all *p-values* > 0.25).

**Supplementary figure 2. Recapitulation of previous heatmaps.**

In a previous study by Pomerantz *et al*. androgen receptor binding sites (ARBS) were identified that were specific for tumor (T-ARBS) and normal (N-ARBS) samples. (A) We reanalyzed the publicly available Pomerantz patient data in our bioinformatics pipeline and with unsupervised clustering were able to recapitulate the segregation of tumor and normal samples, validating our methodology.

**Supplementary Figure 3. Validation and analysis of transfections.**

RT-qPCR was performed on equivalent samples from Figure 2, where LHSAR cells were transfected with FOXA1or GATA2 expression plasmids or c-JUN siRNAs, or appropriate controls. Appropriate overexpression or knock down was observed with each (A-C). The effects on the levels of AR and AR target genes were also quantified (D-F). FOXA1 and c-JUN did not alter expression of AR or AR responsive genes. Overexpression of GATA2 did result in a significant increase in AR directly and also the AR responsive genes FKBP5 and KLK3 (aka PSA). Error bars represent SEM (n=3) and statistical analysis was by a two way t-test. A *p-value* of > 0.05 was considered significant as indicated by *.
